# The Early Sex-Specific Expression of the Fruitless Gene in the Asian Tiger Mosquito *Aedes albopictus* (Skuse) and Its Functional Conservation in Male Courtship

**DOI:** 10.3390/insects16030280

**Published:** 2025-03-07

**Authors:** Marianna Varone, Paola Di Lillo, Katerina Nikolouli, Ayca Eda Özel, Francesca Lucibelli, Gennaro Volpe, Sarah Maria Mazzucchiello, Angela Carfora, Serena Aceto, Giuseppe Saccone, Kostas Bourtzis, Marco Salvemini

**Affiliations:** 1 Department of Biology, University of Study of Naples Federico II, 80100 Naples, Italy; marianna.varone@unina.it (M.V.); paola.dilillo@unina.it (P.D.L.); francesca.lucibelli@unina.it (F.L.); gennaro.volpe2@unina.it (G.V.); sarahmaria.mazzucchiello@unina.it (S.M.M.); angela.carfora@unina.it (A.C.); serena.aceto@unina.it (S.A.); giuseppe.saccone@unina.it (G.S.); 2 Insect Pest Control Laboratory, Joint FAO/IAEA Centre of Nuclear Techniques in Food and Agriculture, Department of Nuclear Sciences and Applications, IAEA Laboratories, 2444 Seibersdorf, Austria; k.nikolouli@iaea.org (K.N.); a.e.ozel@iaea.org (A.E.Ö.); k.bourtzis@iaea.org (K.B.)

**Keywords:** courtship behavior, *fruitless*, *Aedes albopictus*, sex determination, alternative splicing, vector control

## Abstract

The Asian tiger mosquito, *Aedes albopictus*, is an invasive species and a vector for several significant human pathogens. Gaining a deeper understanding of its reproductive biology offers valuable insights into its evolutionary success and may inform the development of sustainable strategies to control its spread. This study presents a comprehensive structural and functional characterization of the *fruitless* gene in *Ae. albopictus* (*Aalfru*), a pivotal regulator of sexual behavior in insects. Through in silico analysis combined with molecular and functional genetics approaches, we identified a high degree of conservation in the *fru* gene structure and its regulation via sex-specific alternative splicing. Differently from *Drosophila*, *Aedes aegypti*, and other dipteran *fruitless* orthologs, *Aalfru* sex-specific regulation starts in 1-day-old embryos, rather than the late larval stage. Functional analysis using embryonic RNA interference (RNAi) demonstrated that, *Ae. albopictus* males with transiently disrupted *fru* expression at the embryonic stage showed significant deficits in adult mating behavior and failed to produce viable progeny. Our findings elucidate the *Aalfru* gene’s molecular organization, developmental regulation, and critical role in courtship behavior, highlighting its importance in male sexual behavior and reproductive success in *Ae. albopictus*.

## Simple Summary

Courtship and mating are key behaviors for the survival and reproductive success of animal populations. In insects, the *fruitless* (*fru*) gene is widely recognized as a central regulator of male mating, orchestrating courtship behavior through sex-specific expression patterns within specialized neural circuits. *fru* orthologs that produce sex-specific isoforms via alternative splicing have been identified across various orders of holo- and hemimetabolous insects, highlighting both the evolutionary conservation of this gene and its ancestral role in courtship regulation within insect taxa. This study focuses on the *fru* ortholog characterization in the Asian tiger mosquito, *Aedes albopictus* (*Aalfru*), and on functional analysis by RNA interference (RNAi)-mediated knockdown to explore its involvement in the genetic control of male courtship behavior.

## 1. Introduction

Courtship in the animal kingdom consists of behavioral displays and actions performed by individuals (often males, but not always) to attract a mate and initiate reproductive pairing. These behaviors, which require the evolution of specialized adaptations in neural, sensory, and motor systems, communicate readiness to mate, assess potential mates’ suitability, and ensure successful mating between compatible partners [1].

Insects, reflecting their remarkable biodiversity, exhibit an extraordinary range of courtship strategies that rely primarily on innate, stereotyped responses to diverse external stimuli. These courtship behaviors can encompass various sensory modalities, including elaborate visual displays, intricate acoustic signals, specific physical gestures, and complex chemical cues [2]. This reliance on varied and often highly specialized sensory channels underscores insects’ evolutionary adaptations in response to selective pressures within their environments, leading to a rich tapestry of species-specific courtship mechanisms across taxa [3,4].

Despite the vast taxonomic diversity of insects, much of our current knowledge regarding the neurogenetic foundations of courtship behavior is derived primarily from studies on *Drosophila melanogaster* [5]. In *Drosophila*, the genes *fruitless* (*fru*) and *doublesex* (*dsx*) are the master regulators in the development and functional activity of neurons that drive sex-specific behaviors. *dsx* is responsible for determining sexual fates in both neural and non-neural cells, contributing to sex-specific physical traits as well as behavior [6]. In contrast, *fru* functions exclusively within the nervous system, shaping the circuits involved explicitly in male courtship and other sexually dimorphic behaviors [7]. *dsx* and *fru* function as terminal regulators within the somatic sex determination cascade of *D. melanogaster*, and their regulation occurs through shared *cis*-regulatory elements. This regulation is achieved for both genes via sex-specific alternative splicing, mediated by a complex containing the serine–arginine-rich female-specific splicing regulator Transformer (TRA) and the non-sex-specific RNA-binding protein Transformer-2 (TRA-2) [8,9].

In *Drosophila*, the functions of *fru* that drive male-specific sexual behavior are primarily mediated by transcripts originating from one of the four identified *fru* promoters, named P1 [2]. *fru*-P1 transcripts undergo sex-specific alternative splicing from the third instar larval stage through adulthood. This results in male-specific transcripts encoding for BTB protein family (Broad-complex, Tramtrack, and Bric-a-brac) transcription factors. Conversely, female-specific transcripts do not produce functional proteins [10]. Transcripts derived from the remaining *fru* promoters (P2, P3, and P4) are present in both sexes but serve distinct roles depending on their timing and tissue of expression. Transcripts produced from promoters P3 and P4 are expressed as early as the embryonic stage and contribute to proper neuronal development [11,12], whereas P2-derived transcripts appear during the pupal stage and are critical for the differentiation of imaginal disc derivatives [10]. These complex promoter and splicing regulations allow *fru* to serve sex-specific and general developmental functions essential to *Drosophila*’s neurogenetic and behavioral systems. Neurogenetic studies have revealed that *fru* helps to establish neurons with sexually dimorphic neurite projections and dendritic branching acting at different levels. At the chromatin regulation level, FRU forms complexes with co-factors, enabling it to function as both an activator or repressor, thereby directing the masculinization or feminization of specific neuronal types [13,14]. At the transcriptional regulation level, genome-wide analyses have identified neuronal development genes directly targeted by FRU, including the *Roundabout* (*Robo*) family, essential for axon pathfinding [15], and *hunchback*, which influences neurite branching [16]. FRU’s role also extends to the proteasomal regulation level, stabilizing the transcription factor Lola in males and preserving male-specific neuron structures and projections [17].

While *Drosophila* provides a valuable model due to its genetic tractability and well-mapped neural circuitry, this focus limits our broader understanding of the molecular and neural mechanisms underlying courtship across other insect taxa. To overcome such limitations, in the past 20 years, orthologs of the *fru* gene have been identified not only in several other dipteran species [18,19,20,21] but also in various orders of holometabolous insects, including Hymenoptera [22], Lepidoptera [23], and Coleoptera [24], as well as in hemimetabolous insects, such as Orthoptera [25] and Hemiptera [26]. These studies have revealed insights into the evolution, structure, and function of the *fru* gene, highlighting the conservation of its regulation via sex-specific alternative splicing in holometabolous insects and a more variable situation in hemimetabolous insects and suggesting an ancestral role in sex determination and sexual behavior.

Within Diptera, of particular interest is the functional study of the *fru* gene in the mosquito *Aedes aegypti* by CRISPR-Cas9 technology to generate *fru* mutant individuals [27]. The study demonstrated that mutant males could not mate, thereby confirming the conserved role of the *fru* gene as a key regulator of male courtship and sexual behavior across insect species. Unexpectedly, the researchers also observed that *fru* mutant males exhibited a strong attraction to live human hosts—behavior absent in wild-type males. This surprising result provided the first evidence that male mosquitoes possess the neural circuitry necessary for host-seeking behavior. The findings further indicated that the functional presence of *fruM* transcripts are essential for repressing this trait in males, effectively keeping it latent. These observations underscored an intriguing evolutionary innovation, wherein a master regulator of male-specific sexual behavior has been repurposed to control the female-specific trait of blood-feeding [28].

In this study, we provide an in-depth structural and functional analysis of the *fruitless* gene in *Ae. albopictus* (*Aalfru*), the Asian tiger mosquito (Diptera, suborder Nematocera). Through an integrative approach combining in silico analyses, molecular techniques, and functional genetics, we identified a highly conserved structural element within the *fru* gene. Our findings further elucidate the gene’s regulation via sex-specific alternative splicing mechanisms initiated during early embryonic stages. Functional analysis using embryonic RNA interference (RNAi) further revealed that male *Ae. albopictus* individuals with disrupted *fru* expression exhibited marked impairments in mating behavior and could not produce offspring. These findings provide a detailed perspective on the molecular organization, developmental regulation, and functional role of *Aalfru*, underscoring its critical influence on male courtship behavior and reproductive success in this relevant vector species [27] and providing insights that may guide the development of innovative sustainable strategies for controlling its spread.

## 2. Materials and Methods

### 2.1. Insect Rearing

Wild-type *Ae. albopictus* used in this study was from Rimini (Italy). Dr. Romeo Bellini established this strain at the Centro Agricoltura Ambiente “Giorgio Nicoli” Srl (CAA, Crevalcore, ITALY), and it has been maintained in the Department of Biology-Federico II University (Naples) since 2019. The mosquitoes were reared under laboratory standard conditions, i.e., 26–27 °C, 60% RH, and 12 h light/12 h dark cycle. Larvae were reared in plastic trays filled with deionized water [29] and provided with TetraMin tropical fish food flakes (Tetra Goldfish granules, Tetra GmbH, Melle, Germany). The adults (sex ratio approximately 1:1) were kept in 32.5 × 32.5 × 32.5 cm or 17.5 × 17.5 × 17.5 cm rearing cages (Bug Dorm, MegaView Science Co., Ltd., Taichung, Taiwan) with constant access to 10% (*w*/*v*) sucrose and females were blood fed on porcine blood twice a week using the Hemotek system (Hemotek Membrane Feeding Systems, Blackburn, UK). Germination papers were provided in adult cages for oviposition. Laid eggs were collected and stored in plastic containers with sufficient humidity (>90% relative humidity) [30] and then transferred to a larval artificial diet (TetraMin) to obtain the hatching. At the Insect Pest Control Laboratory (IPCL), the *Ae. albopictus* Rimini strain was also used for embryonic microinjections, RT-PCRs, and behavioral experiments. Mosquitoes were reared at 27 ± 1 °C, 80% relative humidity, and a 14/10 h day/night photoperiod. Larvae were fed a 4% liquid diet consisting of tuna meal (50%), bovine liver powder (35%), and brewer’s yeast (15%), dissolved in deionized water, while adult mosquitoes were offered a 10% sucrose solution [31]. Blood-feeding was performed twice weekly using porcine blood, and eggs were collected 72 h after the last blood-feeding using moistened oviposition papers (white germination paper, Sartorius Stedium Biotech, Vienna, Austria). The blood used was collected in Himberg, Austria, during the routine slaughtering of pigs in a nationally authorized abattoir, conducted at the highest possible standards strictly following EU laws and regulations.

### 2.2. Preparation of Double-Stranded RNA (dsRNA) with In Vitro Transcription

Template DNA was amplified with *fruitless* male-specific primers (Table 1: *Aalfru_MB_T7+/Aalfru_MB_T7−)* containing T7 promoter sequence (5′-aatacgactcactataggg-3′) at their 5′ ends. The resulting product, after purification from agarose gels using StrataPrep DNA Gel extraction kit (Agilent, Santa Clara, CA, USA), was used as a template to synthesize dsRNA using the MEGAscript RNAi T7 kit (Invitrogen™, Waltham, MA, USA) according to manufacturer’s recommendation. Similarly, a fragment of *GFP* gene was amplified by PCR from the pAct:dCas9 vector (Addgene, Teddington, UK) using *eGFP*-specific primers (Table 1: eGFP_T7+/eGFP_T7−), each containing the T7 promoter sequence at the 5′ end; additionally, dsRNA was synthesized in vitro as previously described.

### 2.3. Embryonic Microinjections of dsRNA

Three to four days after blood-feeding, females were placed in *Drosophila* vials (Thermo Scientific™, Wilmington, NC, USA) containing a wet filter paper. Embryos to be injected were collected after allowing females to oviposit for ≤90 min in a dark environment. Light-grey embryos were aligned, transferred on coverslips with double-sided sticky tape, allowed to desiccate briefly, and covered with Halocarbon oil 27 (Sigma-Aldrich Co., Darmstadt, Germany) [32]. Microinjections were performed at the posterior pole of embryos using a mix of 1 μg/μL of dsRNA (dsRNA_*fru*) and injection buffer (5 mM KCl, 0.1 mM sodium phosphate, pH 6.8). Injections were carried out using the XenoWorks^®^ Digital Microinjector (Sutter Instrument Co., Novato, CA, USA) with QUARTZ 0.7 mm needles with filament, which were pulled using a P-2000 Laser-Based Micropipette Puller (Sutter Instrument Co., Novato, CA, USA). The needle pulling specifications were as follows: HEAT = 750, PULL = 200, VEL = 40, DEL = 145, and FIL = 3. A set of control eggs was injected with dsRNA*_GFP* and kept for hatching in parallel with a set of un-injected eggs laid in the same batch. After injections, oil was washed off the embryos, and the coverslips were covered with wet filter paper and allowed to recover under controlled insectary conditions (26–27 °C; 60% RH) for five to six days [32]. Subsequently, the embryos were hatched to a new tray containing larval diet. This experiment was conducted at the IPCL in Seibersdorf (Austria) in accordance with FAO/IAEA guidelines.

### 2.4. RNA Extraction and cDNA Preparation

Total RNA was isolated from all samples: eggs (E_0–24h_), larvae (L_I-IV_), pupae (P), and adults (M and F) (N = 10), and different tissues: abdomen (A), heads (H), and carcasses (C) (N = 10), following the TRIzol Reagent (Thermo Fisher Scientific, Waltham, MA, USA) protocol. Total RNA from larvae injected with dsRNA (L_IV_) (N = 13 or N= 3) was extracted using the RNeasy Micro Kit (Qiagen, Hilden, Germany). RNA quantification measurements were performed using NanoDrop™ 2000/2000c Spectrophotometers (Thermo Scientific™, Wilmington, NC, USA). Extracted RNA (0.5 µg) was retro-transcribed into cDNA using the LunaScript^®^ RT SuperMix Kit (NEB, Ipswich, MA, USA) according to the manufacturer’s instructions.

### 2.5. RT-PCR and RT-qPCR

RT-PCRs were performed using the LongAmp^®^ Taq DNA Polymerase (NEB, Ipswich, MA, USA) according to the manufacturer’s instructions. Appropriate annealing temperatures and cycle numbers were adjusted empirically for each primer pair. The primer list is shown in Table 1. The primer pair *Aalrp49+/Aalrp49−* was used as positive control [33]. All gene expression levels in this study were determined using a real-time PCR machine, CFX96 Touch Deep Well Real-Time PCR System (Bio-Rad, Hercules, CA, USA). RT-qPCR was conducted with a reaction volume of 20 μL consisting of 10 μL of 2× iQ SYBR Green Supermix (Bio-Rad, Hercules, CA, USA), 1 μL cDNA template, 0.5 μL forward and reverse primers (10 μmol/L), and 8 μL RNase-Free ddH2O. RT-qPCR was performed under the following conditions: denaturation at 95 °C for 2 min, followed by 40 cycles of dissociation at 95 °C for 15 s, annealing at 58 °C for 30 s, and extension at 72 °C for 30 s. Expression levels were calculated using the 2^−ΔΔCt^ method with a triple technical for each sample [34], all data normalized to the housekeeping gene expression *rp49*.

### 2.6. Sequence Analysis

Based on the in silico assembled sequence, a set of primers were designed to amplify the complete *Aalfru* gene (*Aalfru_M2+/Aalfru_C3−*; *Aalfru_C3+/Aalfru_ZnfC−*; *Aalfru_C5+/Aalfru_ZnfA−*; and *AalfruC5+/Aalfru_ZnfB−*). The sequence of *Aalfru* was produced using the Mix2Seq Kit (Eurofins genomics, Ebersberg, Germany). The sequence reactions were assembled into Eurofins tubes using 10 µM *fruitless*-specific primers (see Table 1) and using as a template the RT-PCR gel-purified *Aalfru* product (StrataPrep DNA Gel extraction kit—Agilent, Santa Clara, CA, USA).

### 2.7. Behavioral Assays

All behavioral experiments were carried out under insectary standards conditions, i.e., 26–27 °C, 60% RH, and 12 h light/12 h dark cycle. To examine whether *Aalfru*-knockdown males exhibited altered mating behavior, 1 male (who had never met a female and had never experienced mating) and 5 virgin females (who originated from the same cohort that emerged on the same day) were crossed (pupae were sexed and separated into individual tubes until emergence to obtain virgin individuals).

A total of three types of crosses were produced:

(a) 1 wild-type male × 5 wild-type females (WT);
(b) 1 *GFP*-knockdown male × 5 wild-type females (*GFP*);
(c) 1 *fru*-knockdown male × 5 wild-type females (*fru*).

Eight replicates were conducted for the WT and *fru* crosses and four replicates for the *GFP* crosses. The crosses were realized in 17.5 cm × 17.5 cm × 17.5 cm cages (BugDorm-4E1515).

The crosses (a), (b), and (c) were observed to analyze the following aspects:

(1) Courtship and mating;
(2) feeding;
(3) fecundity.

(1) The fraction of time spent by the male on various aspects of courtship and mating behavior was recorded through direct observation using manual scoring under controlled laboratory conditions. In each observation session, cages were monitored starting from the moment males and females were introduced, with a total observation time of 30 min per session conducted twice per day, in the early morning and late afternoon.
(2) Following a three-day mating period, the cages were examined for feeding behavior. Mosquitoes had constant access to 10% (*w*/*v*) sucrose, and warm porcine blood was offered at the top of the cages for 30 min. Feeding was assessed based on visual observation of the abdomen of the male and female mosquitoes.
(3) Three days after the blood meal, plastic cups containing deionized water and lined with germination paper were provided in each cage for 48 h. Eggs were counted and examined under an optical stereomicroscope.

### 2.8. Statistical Analysis

Experimental data were analyzed using GraphPad Prism 9.0 (GraphPad Software, San Diego, CA, USA). The expression pattern analysis was performed by analysis of variance (one-way ANOVA test) followed by Dunnett’s multiple comparisons test or Tukey post hoc test). Data collected as a percentage of the total are shown as mean ± SEM. Details of statistical methods are reported in the figure legends.

## 3. Results and Discussion

### 3.1. The Molecular Characterization of the Aalfru Gene

**Figure 1:**
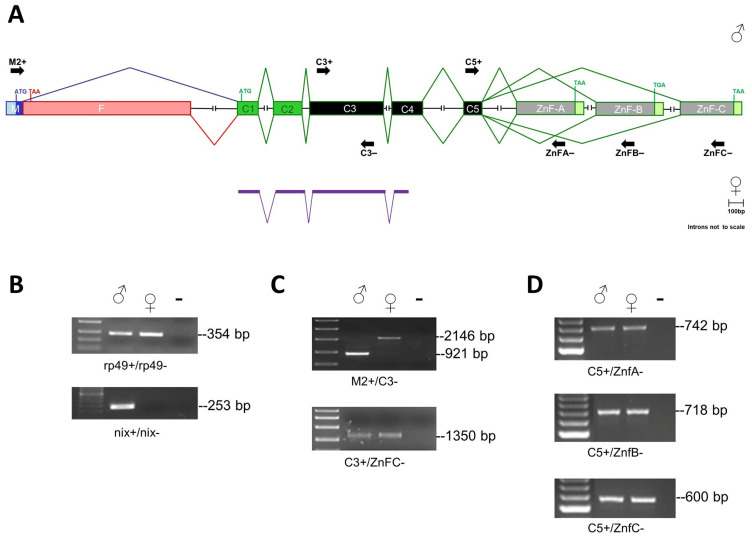
The *Aalfru* gene structure. (**A**) Schematic representation of the *fru* gene in *Ae. albopictus* (introns not to scale). In purple, are the exons belonging to the predicted AALF007440 gene of the *Ae. albopictus* AaloF1 reference genome. The translational start (ATG) and stop (TGA, TAA) sites are indicated. The exons C1 and C2 encode for the BTB domain; the exons C3, C4, and C5 encode for the connecting region; the terminal exons Znf-A, Znf-B, and Znf-C encode for the type A, B, and C zinc finger domains, respectively. The sex-specific region is divided into two sub-regions: male- (M in blue) and female-specific (F in pink) portions, which are alternatively spliced according to sex. (**B**) The RT-PCR amplification with *Aalrp49* and *AalNix* positive controls. The first lane left is a 100-bp ladder (NEB). The *Aalrp49* primer pairs span a 113-bp long intron of the *rp49* gene (genomic amplicon size 467 bp; cDNA amplicon 354 bp). The *Nix* gene is a male-specific positive control. (**C**) The RT-PCR amplifications of *Aalfru* sex-specific and common cDNA fragments on sexed adult *Ae. albopictus* mosquitoes. The first lane left is the High Range ladder (NEB). (**D**) RT-PCR amplifications of *Aalfru* terminal cDNA fragments encoding for zinc finger domains on sexed adult *Ae. albopictus* mosquitoes. The first lane on the left is a 100-bp ladder. Primers used in the PCR amplifications of (**B**–**D**) panels are indicated as short black arrows in (**A**).

In insects, the *fru* gene exhibits a complex genomic organization, generating transcript isoforms by alternative promoters, mutually exclusive terminal exons, and sex-specific alternative splicing [2]. In most species, including mosquitoes, the gene region responsible for sex-specific alternative splicing, transcribed from the P1 promoter, is located at a large genomic distance from the gene’s first exon, C1, which is common to all *fru* transcripts and encodes the DNA-binding BTB domain [2]. As a result, automated gene prediction systems frequently fail to produce a complete *fru* gene model that includes all exons. In the *Ae. albopictus* AaloF1 reference genome, available at the Ensembl Metazoa genome browser https://metazoa.ensembl.org/ (accessed on 2 February 2024), a partial *fru* gene model is present (AALF007440, located in the supercontig JXUM01S001695), including only the first four common exons (Figure 1A). To reconstruct the complete genomic organization of the *fru* gene in *Ae. albopictus*, we conducted a TBLASTN analysis of the AaloF1 genome using as virtual probes the *Ae. aegypti* P1-FRU-ZnF-C male-specific transcript (GenBank acc. Num.: JX186753), and the putative terminal exon sequences encoding the ZnF-A and ZnF-B domains [21]. We identified the following: (1) a sex-specifically regulated exon, located in a different supercontig (JXUM01S000018) and divided into two subregions (male- and female-specific portions); (2) five common exons (C1-C2-C3-C4-C5); (3) three putative alternative zinc finger encoding exons, corresponding to zinc finger type A, B, and C (Figure 1A). Using primer pairs specific for the *Aalfru* exons identified in silico, we performed RT-PCR experiments on RNA samples extracted from adult-sexed mosquitoes, confirming their presence in the transcriptional units of both sexes (Figure 1C,D). At first, we amplified a reference gene, *rp49* (Table 1: *Aalrp49+/Aalrp49−*), as positive control and the male-specific *AalNix* gene (Table 1: *Aalnix+/Aalnix−*) to confirm the genetic sex of mosquitoes (Figure 1B).

Furthermore, RT-PCR analysis using a forward primer located in putative exon M and a reverse primer located in the common exon (*Aalfru*_M2+/*Aalfru*_C3−) produced male-specific (921 bp) and female-specific (2146 bp) cDNA amplification in adults, confirming the regulation of the *fru* gene by sex-specific alternative splicing also in *Ae. albopictus* (Figure 1C). The cDNA products were sequenced (Supplemental Materials File S1) and the alignment of their sequences with the AaloF1 genome led us to define an updated *Aalfru* genomic organization, represented in Figure 1A.

**Figure 2:**
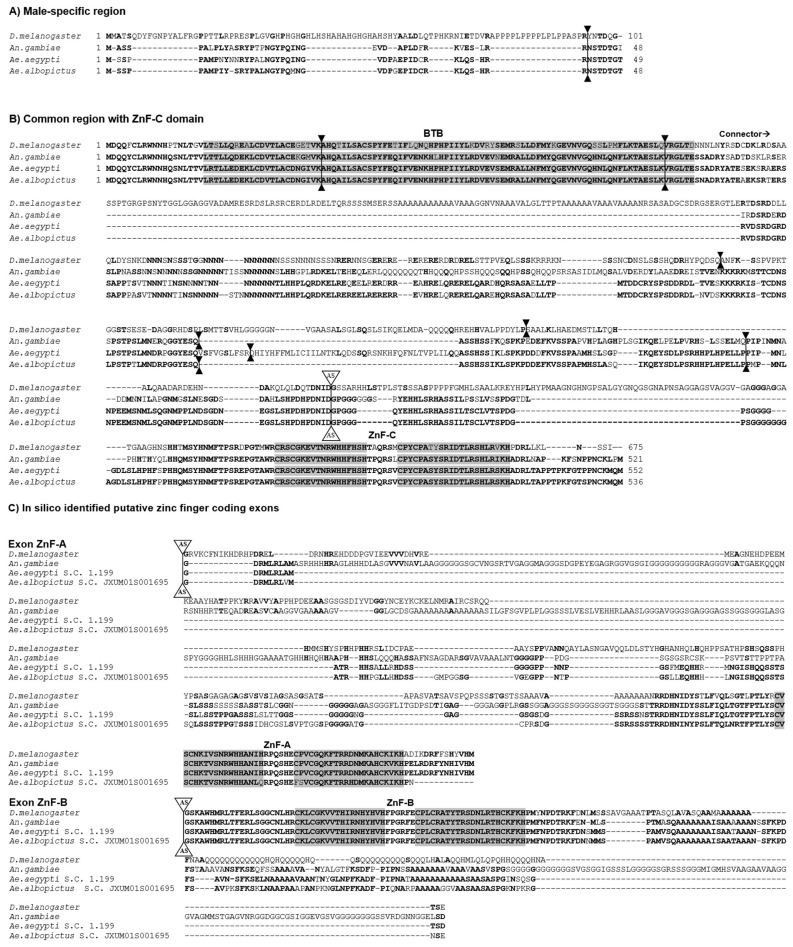
The sequence alignment of the FRU protein isoforms of *D. melanogaster*, *An. gambiae*, *Ae. aegypti*, and *Ae. albopictus*. The conserved BTB domain and zinc finger domains are boxed in grey. The bold letters indicate amino acid identity among at least two species. The intron positions are indicated by solid triangles and the position of the alternative splicing site is indicated by AS white triangles. Gaps were introduced in the alignments to maximize similarity. The sequences are divided into (**A**) male-specific N-terminal portion encoded by P1 transcript; (**B**) common portion of the gene including the BTB domain, the connector region, and the zinc finger type C domain; and (**C**) putative in silico identified zinc finger of type A and B domains of *Ae. albopictus* aligned with the homologous domains of *D. melanogaster*, *An. gambiae*, and *Ae. aegypti*.

Next, we compared the putative *Aal*FRU protein isoforms with the FRU isoforms of *D. melanogaster*, *An. gambiae*, and *Ae. aegypti* (Figure 2). The alignments revealed the high conservation of the BTB, ZnF-A, ZnF-B, and ZnF-C domains between species (Figure 2B,C), but a very low similarity between the connector and male-specific N-terminal domains (Figure 2A).

### 3.2. The Developmental Expression Analysis of the Aalfru Gene

**Figure 3:**
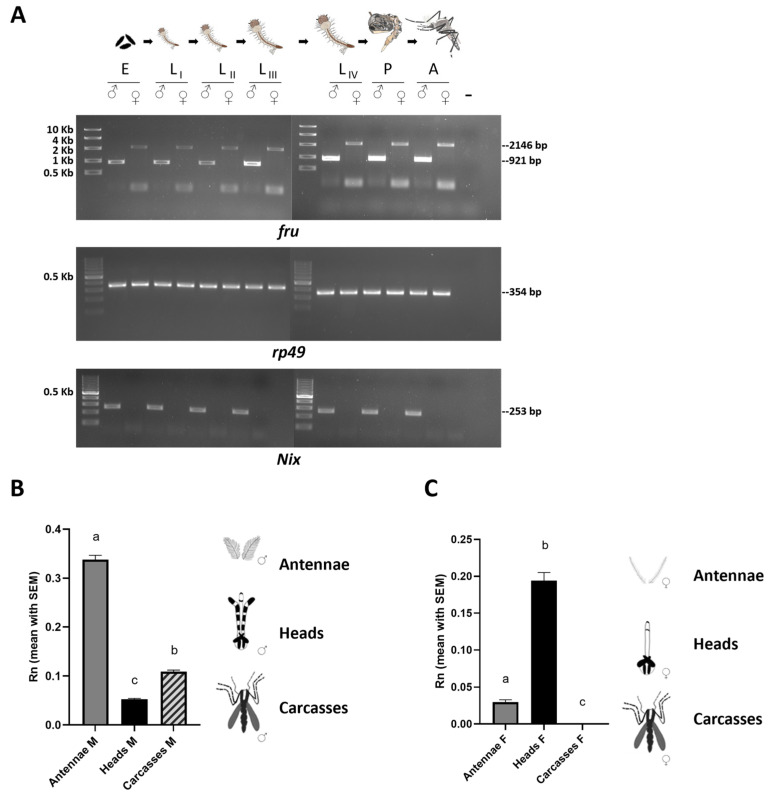
The developmental and spatial expression analyses of the *fru* gene in *Ae. albopictus*. (**A**) The first lane left of each panel is a High-Range ladder or 100-bp ladder (NEB). RT-PCR amplifications of *Aalfru* were performed with M2+/C3− primer pair (for sex-specific adult isoforms) on the following sexed samples: E = 0–24 h embryos; L_I_ = first instar larvae; L_II_ = second instar larvae; L_III_ = third instar larvae L_IV_ = fourth instar larvae; P = pupae; and A= adults. The *Aalrp49* and *AalNix* were used as a positive control and male-specific control, respectively. (**B**,**C**) The tissue-specific transcription profile of *Aalfru* in males and females. The spatial transcription on male and female antennae, heads, and carcasses (minus heads) sampled by qRT-PCR. The x-axis indicates the sample ID and the y-axis shows the relative expression value obtained by qRT-PCR. The internal reference gene was the *Ae. albopictus* ribosomal protein 49 (*Aalrp49*). (**B**) The error bars represent the SEM (N = 10) (one-way ANOVA test, *p*-Value <0.0001). Tukey post hoc test: ‘a’ vs. ‘c’ and ‘a’ vs. ‘b’ <0.0001; ‘b’ vs. ‘c’ 0,0009); (**C**) The error bars represent the SEM (one-way ANOVA test, *p*-Value <0.0001). Tukey post hoc test: ‘a’ vs. ‘b’ and ‘a’ vs. ‘c’ <0.0001; ‘a’ vs. ‘c’ 0,0482).

In the mosquito *An. gambiae*, the analysis of the sex-specific splicing of the *fru*-P1 transcripts has been conducted exclusively at the adult stage. This analysis revealed, for the first time, the key conservation of the sex-specific alternative splicing mechanism observed in *D. melanogaster*, despite the approximately 250 million years of evolutionary divergence between these two dipteran lineages [18]. In *Ae. aegypti*, the RT-PCR analysis on RNA samples extracted from mixed-sex developmental stages, ranging from embryos to pupae, and on sex-separated adult male and female samples, indicated the presence of sex-specific *fru*-P1 transcripts starting from the third instar larval stage. This is consistent with observations in *D. melanogaster* [21,35]. In the present investigation, we examined the sex-specific expression patterns of *Aalfru*-P1 transcripts in *Ae. albopictus*. To achieve this, we conducted RT-PCR analysis on RNA samples extracted from various sexed developmental stages including embryos (0–24 h), larvae (L_I_, L_II_, L_III_, and L_IV_), pupae (P), and adults (M and F). As internal reference markers, we employed the *rp49* gene, which exhibits constitutive expression in *Ae. albopictus* [33], and the male-specific *Aalnix* gene, utilized to identify the sexual karyotype of the developmental samples [36] (Figure 3A).

Our RT-PCR analysis on sexed individuals revealed that the *Aalfru* gene produces sex-specific transcripts already in 0–24 h old embryos and all other developmental stages, (Figure 3A). This suggests a potential role for the *fru* gene in establishing a dimorphic state in the central nervous system as early as the initial stages of brain development in mosquito larvae. While many dipteran species undertake protracted metamorphosis, during which the adult nervous system forms through the widespread restructuring and substitution of larval neurons, in most mosquitoes, including *Ae. aegypti* and *An. gambiae*, pupae can complete metamorphosis in about one day [26]. Such a rapid metamorphic transition likely limits the extent of neuronal structure reconfiguration, suggesting that a substantial number of larval neurons are conserved into the adult stage in mosquitoes, as observed with some serotonergic neurons that have been shown to persist in *Ae. aegypti* from the larval through to the adult brain [37,38]. We can hypothesize that such an early expression of *fru*-P1 sex-specific isoforms in *Ae. albopictus* may play a role in the development of sex-specific differences in the formation of neural circuits underlying innate behaviors, beginning as early as the larval stage. These differences may then be preserved into the adult stage.

We then performed a tissue-specific expression profiling of the *Aalfru* gene in 2-day-old adult males and females by quantitative RT-PCR using a primer pair targeting both male- and female-specific *Aalfru* P1 transcripts (Figure 3B,C). In males, the *fru* transcripts were detectable in the antennae, head, and carcasses with a significantly higher transcription (*p* < 000.1) in the male’s antennae and a lower level of transcription in the head and carcasses (Figure 3B). The antennae assume a pivotal role in the olfactory and acoustic behaviors of mosquitoes. In male mosquitoes, the antennae play a crucial role in locating females during mate seeking. Male antennae are highly sensitive to the sound vibrations produced by the wingbeats of females and allow males to detect and amplify these frequencies, facilitating the recognition of and orientation toward a female in flight [39]. Additionally, the antennae are equipped with olfactory sensors that enable males to detect chemical signals released by females [39]. The specific high transcription of *fru* within the male mosquito’s antennae strongly suggests its involvement in the modulation of acoustic and olfactory sexual behavior in the Asian tiger mosquito.

The tissue-specific transcriptional profiling of the *Aalfru* gene in female tissues revealed that there is a significant *Aalfru* (*p* < 0.0001) expression in the heads and a low expression in antennae while no expression at all has been detected in carcasses (Figure 3C). The presence of *Aalfru* P1 transcripts in male carcasses and their complete absence in female carcasses could be linked with the presence of male-specific muscles, such as the Lawrence muscle (MOL), in *Ae. albopictus*. Gailey and colleagues [18] showed the presence of a MOL-like male-specific muscle in the abdomen of *An. gambiae* adult males and that the *An. gambiae* FRUMC isoform is sufficient to induce the formation of the male-specific MOL in *D. melanogaster* transgenic female flies and the rescue of MOL development in mutant males. However, to date, no information about the MOL is available for *Aedes* mosquitoes, representing an interesting topic to be addressed in the future.

### 3.3. The Conservation of the Aalfru Genomic Organization and Sex-Specific Splicing Regulation

**Figure 4:**
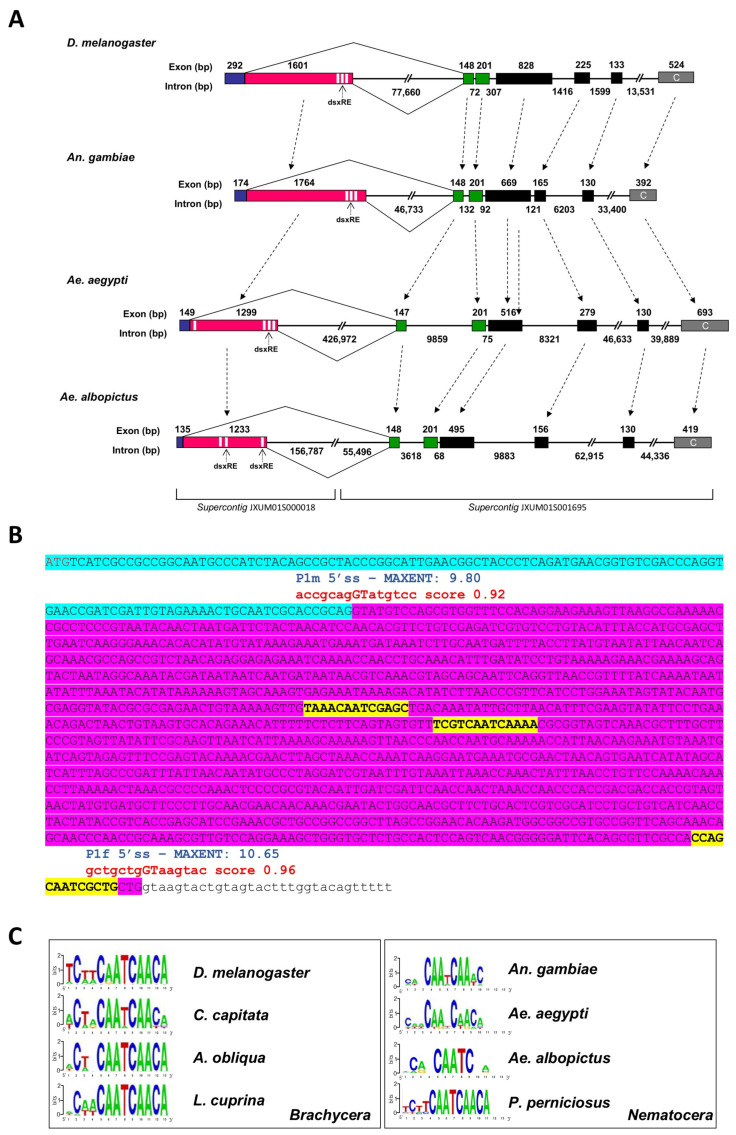
A comparative scheme of *D. melanogaster*, *An. gambiae*, *Ae. Aegypti*, and *Ae. albopictus* gene structures. (**A**) The portions of *fru* genes, starting with the sex-specific regulated region and ending with the ZnF-C domain encoding exon, are reported. The male-specific and female-specific exons are represented as blue and pink boxes, respectively. The green boxes represent the non-sex-specific exons encoding the BTB domain and the black boxes represent the connector region of FRU proteins. The terminal grey boxes represent the ZnF-C domain encoding exons. The white sequences represent TRA/TRA-2 binding sites. The *Ae. albopictus fru* gene is located in supercontigs JXUM01S001695 and JXUM01S000018 of the AaloF1 reference genome and on chromosome 1 of the AalbF5 genome assembly (GCF_035046485.1). (**B**)The *fru-P1* sex-specific region. The azure sequence indicates the male-specific exon; the purple sequence indicates the female-specific exon. The red are the predicted splicing donor sites. The blue are the P1m 5′ss and P1f 5′ss MAXENT scores. The yellow are the putative TRA/TRA-2 binding sites. (**C**) The WebLogo consensus sequence of the putative TRA/TRA-2 binding sites identified in *dsx* and *fru* genes of Dipteran Brachycera and Nematocera species. Within Nematocera, only in the *P. perniciosus* sand fly species it is possible to define a clear TRA/TRA-2 binding sites consensus sequence.

In *Ae. aegypti*, the *fru* gene spans an extensive genomic region of approximately 533 kb, considerably larger than the 130 kb long *fru* gene in *Drosophila* and the 90 kb long *fru* gene in *An. gambiae* [21]. The *Aalfru* gene comprises eight exons and seven introns with substantial length variability. It spans a region of approx. 335 kb in the AaloF1 genome assembly (Figure 4A). We searched in the recently released *Ae. albopictus* AalbF5 chromosome-level genome assembly, available at NCBI website (GCF_035046485.1), and we identified a conserved *fru* locus, with conserved exon–intron organization, located on chromosome 1 and spanning a 587 kb long genomic region (unpub. res.). This highlights how the organization of the *fru* gene within a very large genomic locus is a conserved feature in the *Ae. aegypti* and *Ae. albopictus species*.

To investigate the evolutionary trajectory of *fru* gene organization, we compared the gene structure among *Drosophila*, *Anopheles*, *Ae. aegypti*, and *Ae. albopictus* (Figure 4A). The five non-sex-specific exons in *Aalfru* (C1-C2-C3-C4-C5) show structural similarity to the corresponding exons in *Drosophila*, *An. gambiae*, and *Ae. aegypti fru* genes, with the highest conservation observed in exons C1 and C2, where the encoded amino acid sequences are essentially identical across species. Exons C3, C4, and C5 display greater variability in size and amino acid composition (Figure 2). Additionally, exon P1 encodes a conserved male-specific N-terminal domain and exhibits sex-specific alternative splicing regulation, as seen in the other *fru* orthologues.

We then proceeded to analyze the genomic region where the exon *fru*-P1 is located in *Ae. albopictus* to better understand the structural organization and potential regulatory elements within this region that govern its sex-specific splicing. Exon–intron boundaries were predicted by using the Berkeley BDGP Splice Site Prediction Tool with default parameters http://www.fruitfly.org/seq_tools/splice.html (accessed on 2 February 2024); prediction scores are indicated in red in Figure 4B) and then confirmed by using male- and female-specific *fru*-P1 transcripts vs. genome alignments. The *Aalfru*-P1 transcripts undergo sex-specific alternative splicing from the embryonic stage to adulthood. Similar to what has been observed in *D. melanogaster* and *Ae. aegypti*, the *fru* gene in *Ae. albopictus* features two canonical 5′ donor splice sites (5′ss) in the sex-specifically regulated region [21]. These sites exhibit a conserved sequence matching the eukaryotic 5′ss consensus (MAG/GTRAGT; M = A or C and R = A or G) (Figure 4B). In vivo, these two 5′ss function as alternative sex-specific splicing sites (P1m 5′ss and P1f 5′ss). To assess the intrinsic strength of the two sex-specific 5′ ss, independently of additional flanking regulatory signals, we applied the MaxEntScan algorithm [21,40]. The results shown in Figure 4B corroborated previous findings in the *Ae. aegypti fru* gene, also indicating that the *Aalfru* female-specific P1f 5′ss (MaxEntScan score = 10.65) seems to be stronger than its male-specific P1m 5′ss counterpart (MaxEntScan score = 9.80) suggesting the existence of a mechanism that represses the P1f 5′ss usage in the male sex.

The sex-specific regulation of *fru* has been extensively studied in *D. melanogaster*, where TRA and TRA-2 splicing regulators play a pivotal role in promoting female-specific splicing. This regulation is mediated by the binding of TRA and TRA-2 proteins to *cis*-regulatory elements, known as TRA/TRA-2 binding sites, which are present in multiple copies within the *fru* and *dsx* mRNAs [41,42]. These binding sites are highly conserved across the *fru* and *dsx* genes of many dipteran species within the Brachycera suborder [8,43]. In contrast, within the Nematocera suborder of Diptera, highly conserved TRA/TRA-2 binding sites have been identified only in the sex-determination genes of sandflies (Figure 4C). This suggests a conserved role for TRA and TRA-2 proteins in regulating sex-specific splicing and controlling sex determination in these species [20,44], and the evolution of different mechanisms and regulators in other Nematocera species, including mosquitoes.

Similar to *Ae. aegypti* and *An. gambiae*, the *fru*-P1 region in *Ae. albopictus* lacks highly conserved TRA/TRA-2 binding sites. Notably, the analysis of this region in *Ae. albopictus* revealed an even greater level of divergence. We identified three putative TRA/TRA-2 binding site sequences with an even higher level of sequence degeneration compared to what has been observed in *Ae. aegypti* and *An. gambiae* (Figure 4A,B). Additionally, while the positions of the putative TRA/TRA-2 binding sites in these two species are conserved relative to *Drosophila*, with three sequences located near the P1f 5′ss, and only in *Ae. aegypti* an additional putative sequence found near the P1m 5′ss, the positions of the three sequences identified in *Ae. albopictus* are also divergent, with two of these sequences located in the center of the female-specific region, and only one found near the P1f 5′ss (Figure 4C). These findings suggest that additional elements, such as the genomic context, flanking intronic sequences, and distinct upstream regulatory proteins, likely play a role in modulating the functional activity of *fru* sex-specific splice sites, thereby enabling their precise sex-specific utilization.

### 3.4. The In Vivo Functional Analysis of the fru Gene in Ae. albopictus by RNAi Knockdown

**Figure 5:**
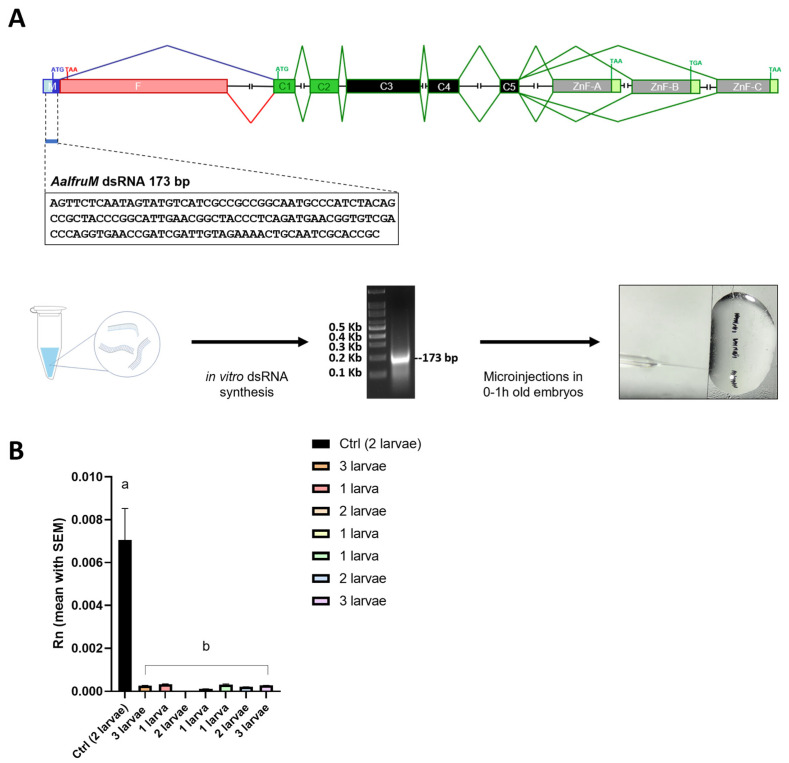
The RNAi knockdown analysis of the *fru* gene in *Ae. albopictus*. (**A**) The schematic representation of the RNAi knockdown analysis. The first lane left is a 100 bp ladder (NEB). The 133 bp long dsRNA synthesized in vitro, targets the M exon of the sex-specifically regulated *Aalfru* gene. This region is present in both the male- and female-specific *Aalfru* transcripts. (**B**) The expression level of the *Ae. albopictus fru* gene in larvae subjected to *AalfruM* interference at the embryonic stage. The larvae were analyzed as single specimens or as a pool of two or three larvae, at the third instar larval stage. The x-axis indicates the sample ID and the y-axis shows the relative expression value obtained by qRT-PCR. The internal reference gene was the *Ae. albopictus* ribosomal protein 49 (*Aalrp49*). The error bars represent the SEM. One-way ANOVA was used to assess differences among the control group and RNAi groups (*p*-Value < 0.0001), Dunnett’s multiple comparisons test: ‘a’ vs. ‘b’ <0.0001. Eight wild-type males, four males developed from embryos injected with GFP dsRNA, and eight males developed from embryos injected with *AalfruM* dsRNA were individually crossed with five wild-type females, resulting in a total of twenty mating experiments (Figure 6A). Before cross set-up, all males were visually inspected under a stereomicroscope for abnormalities in sexual phenotypes, including antennae structure, body size, and genitalia. No notable physical abnormalities were observed. To evaluate the effects of the *Aalfru* knockdown on male sexual behavior, we conducted mating assays by observing the formation of mating pairs within each cage and recording the duration of copulation (Figure 6C). A significant difference in mating duration was noted between control males (wild-type and GFP dsRNA-injected) and *Aalfru*-knockdown males when paired with wild-type virgin females. The mating time for *Aalfru*-knockdown males was substantially reduced, averaging approximately 3 s, compared to an average duration of 20 s for the wild-type and GFP control males (Figure 6C). These results suggest that the suppression of *Aalfru* expression profoundly impacts male sexual behavior, likely impairing their ability to sustain normal mating interactions. This highlights the role of *Aalfru* in regulating the key aspects of male reproductive behavior in *Ae. albopictus*.

To investigate the in vivo function of the *Aalfru* gene, we performed the RNAi knockdown of *fru*-P1 transcripts by microinjecting dsRNA molecules into embryos at the one-hour developmental stage. The dsRNA molecules were specifically designed to target the 5′ region of *fru*-P1 transcripts in both sexes (Figure 5A). Approximately 1400 embryos were injected with these *fru*-specific dsRNAs, while as a negative control, about 500 embryos were injected with dsRNAs targeting the exogenous Green Fluorescent Protein (GFP) gene, absent in the genome of *Ae. albopictus* (Table 2). In both experimental and control groups, we observed high levels of embryo mortality, which we attributed to the microinjection procedure itself and the specific conditions of the *Ae. albopictus* strain utilized for embryo production. Similar mortality rates (98%) were also recorded in additional microinjection tests performed with injection buffer only.

A total of 22 *Aalfru* knockdown larvae were obtained following dsRNA injection. Of these, 13 were selected for further analysis to assess the RNAi-mediated reduction in *fru*-P1 transcript levels. From the control group, seven larvae were obtained, and three were chosen as reference samples for comparison. Using qRT-PCR, we observed a significant reduction (*p* < 0.0001) of more than 90% of *Aalfru* mRNA levels in all larvae tested. This included the individual analysis and pooled analyses of 2–3 larvae at the third instar larval stage, compared to the GFP control group (Figure 5B). These results confirmed the successful knockdown of *fru*-P1 transcripts through embryonic RNAi, demonstrating a significant reduction in expression levels that persists up to the fourth instar larval stage.

In insects, embryonic RNAi is primarily used to study the function of genes involved in early embryonic development and cellular differentiation, rather than genes that play roles in post-embryonic development. This is because the injection of dsRNA into embryos produces a transient effect in the cells, which is not passed on to the next generation. As a result, genes expressed during the later stages of development are generally not easily inactivated using this approach [45,46]. Our developmental expression analysis of the *Aalfru* gene revealed a very early sex-specific expression, starting from the embryonic stage till adulthood. This suggests that this gene may have an early role in establishing sex-specific larval neuron connections in the central nervous system that could persist into adulthood. We tested this hypothesis by analyzing the mating behavior, fertility, and feeding behavior of males developed from dsRNA-injected embryos.

**Figure 6:**
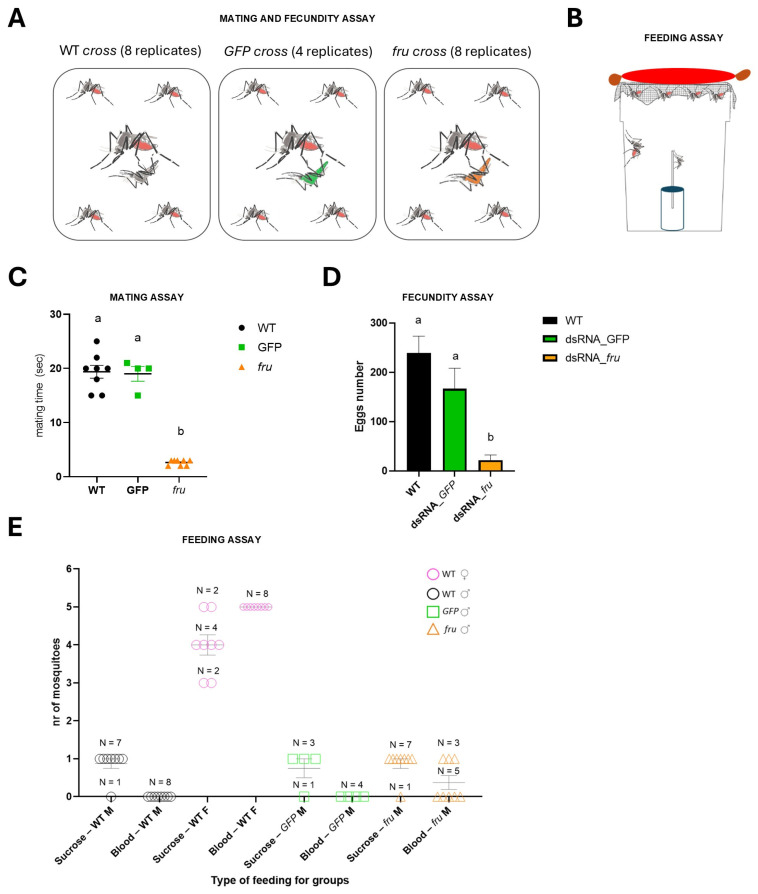
The mating, fecundity, and feeding assays of the RNAi-knockdown *fru* males. (**A**) The mating and fecundity assay schematic. (**B**) The feeding assay schematic. (**C**) The mating time for the three types of crosses is indicated in the A panel. The one-way ANOVA test, *p*-Value < 0.0001. Dunnett’s multiple comparisons test: ‘a’ vs. ‘b’ < 0.0001. (**D**) The egg numbers for the three types of crosses as indicated in the A panel. The one-way ANOVA test, *p*-Value 0.0004. Dunnett’s multiple comparisons test: ‘a’ vs. ‘b’ < 0.0001. The different letters above bars (’a’ vs. ’b’) mean significant differences between the different groups. (**E**) The scatter dot plot shows the number of mosquitoes that fed on sucrose or blood in different groups: wild-type male and female (WT M, WT F), *GFP*, and *fru* males (*GFP* M, *fru* M). Each dot represents an individual cross. The horizontal bars indicate the mean ± standard error of the mean (SEM). N represents the number of crosses (panel A) observed for each group.

To evaluate mating success, we then measured fecundity by collecting and counting the eggs laid by females from each cross over three days. A statistically significant difference in the average number of eggs was observed between the *Aalfru*-knockdown crosses and the control groups (WT and GFP dsRNA-injected males) with a reduction of more than 90% (Figure 6D). Specifically, females mated with *Aalfru*-knockdown males laid significantly fewer eggs compared to those mated with control males. Notably, none of the eggs from the *Aalfru*-knockdown crosses produced viable larvae, indicating that the eggs were non-viable. This suggests that despite the brief mating interactions observed between *Aalfru*-knockdown males and wild-type females, successful reproduction did not occur. The lack of fertilization in these crosses indicates that the suppression of *Aalfru* impairs the males’ ability to effectively fertilize eggs, further underscoring the critical role of *Aalfru* in male reproductive competence and overall mating success.

Basrur et al. (2020) used CRISPR-Cas9 to create *fru* mutant *Ae. aegypti* males, showing they were not only unable to mate, confirming *fru* as a key regulator of male courtship across insects, but also that they were strongly attracted to live human hosts, a behavior not present in wild-type males [28]. This revealed that males possess neural circuits for host-seeking behavior, normally repressed by *fruM*, highlighting an evolutionary adaptation where a male-specific regulator also controls a female-specific trait—blood-feeding [28]. To verify whether in *Ae. albopictus*, the *fru* gene could also be involved in the repression of the female-specific blood-feeding behavior as in *Ae. aegypti*, a feeding assay (Figure 6B) was developed where both male and female mosquitoes were provided with access to either a sucrose solution or a blood meal source. Both *Aalfru*-knockdown and *GFP*-knockdown males exhibited normal feeding behavior on the sucrose source, consistent with the behavior observed in wild-type males and females. To test blood-feeding responses, a warm blood-filled sausage, which had been rubbed on the operator’s arm to transfer odor molecules mimicking human skin scent, was placed atop the cages. Wild-type females fed on the blood sausage, as expected. Interestingly, three out of eight *Aalfru*-knockdown males also exhibited attraction for the blood source, which was completely absent in all *GFP*-knockdown males and wild-type males (Figure 6E). These three *Aalfru*-knockdown males actively explored the area near the sausage, probing the surface of the sausage membrane with their mouthparts, mimicking the blood-feeding behavior typically observed in females. This suggests that *Aalfru* knockdown disrupts the male-specific feeding program, partially inducing female-like blood-feeding behavior.

## 4. Conclusions

This study underscores the crucial role of the *fruitless* (*fru*) gene in regulating male-specific behaviors in *Ae. albopictus*. Through detailed structural and functional analyses, we revealed a high degree of conservation in *Aalfru* gene organization and its sex-specific alternative splicing mechanisms. In addition, we showed that the *Aalfru* gene exhibits an early sex-specific expression, starting from the embryonic stage, a novel feature not yet reported for any dipteran species.

An open question remains about the upstream splicing regulators of *fru*, as well as of the *dsx* gene in *Aedes* mosquitoes. The absence of evidence of the direct interaction between *AalNix* protein and the pre-mRNA of *Aaldsx*, combined with evidence supporting an indirect regulation of splicing and the presence of potential splicing enhancer elements, strongly suggests the existence of an as-yet unidentified intermediate regulator within the sex determination cascade of *Ae. albopictus* [47]. This hypothetical regulator may act as a mediator between upstream signals, such as those involving *AalNix*, and the downstream splicing machinery responsible for producing sex-specific isoforms of *Aaldsx* and *Aalfru* orthologs. Further investigation into this intermediate factor is essential to fully elucidate the molecular mechanisms driving sex determination in this species, as its identification could bridge critical gaps in our understanding of how upstream genetic signals are translated into the regulation of alternative splicing events.

Our transient embryonic RNA interference-mediated knockdown of *fru* in *Ae. albopictus* demonstrated that its disruption leads to significant impairments in male courtship and mating behavior, ultimately resulting in reproductive failure. Our results suggest an essential role of the *fru* gene in the early formation of sex-specific neuronal connections in mosquitoes. The microinjections performed on embryos immediately affected neurobiological development, and these changes were found to persist in adulthood. Our findings suggest that the neuronal structures formed during the early larval stages are crucial for the sexual behavior and adaptation of adult mosquitoes. Furthermore, the unexpected observation of female-like blood-feeding behavior found in *Ae. aegypti* also seems to be conserved in *fru*-knockdown *Ae. albopictus* males, confirming an intriguing evolutionary repurposing of *fru*, which functions to repress female-specific traits in males. In the future, it would be highly valuable to identify the downstream genes in the pathway of the *fru* gene by utilizing mutant lines generated through CRISPR-Cas9 approaches and combined with transcriptomics. This would provide deeper insights into how this gene contributes to the establishment of sex-specific behavioral patterns.

In conclusion, our findings not only provide insights into the genetic control of determination in *Ae. albopictus* but also open new avenues for developing innovative, gene-targeted strategies for Asian tiger mosquito population management and vector control based on the interference with the courtship and mating behaviors, as recently proposed for other mosquito species using the *dsx* gene splicing manipulation [48,49].

## Figures and Tables

**Table 1:** Primers list.

Name	Sequence (5′-3′)	Purpose
*Aalrp49+*	GACGAAGAAGTTCATCCGCC	cDNA amplification
*Aalrp49−*	GTTCTGCTGCGAGCGCAG	cDNA amplification
*Aalnix+*	GTTGTTCGTTACAGACTGATG	cDNA amplification
*Aalnix−*	CAAAGCTAATGTAAACCATGAC	cDNA amplification
*Aalfru_M2+*	GAACGGCTACCCTCAGATGA	cDNA amplification and sequencing
*Aalfru_C3−*	ACCTGCGATTCGTATCCACC	cDNA amplification and sequencing
*Aalfru_C5+*	ACATGCCTCCGCTGAACGA	cDNA amplification and sequencing
*Aalfru_ZnfA−*	CCGTTGTTTGTTCCGGGC	cDNA amplification and sequencing
*Aalfru_ZnfB−*	AGCACTGCTCGACTGGC	cDNA amplification and sequencing
*Aalfru_ZnfC−*	CGGTAGGTGTCGCCTTGTT	cDNA amplification and sequencing
*Aalfru_real+*	CGGCAATGCCCATCTACAG	qRT-PCR
*Aalfru_real−*	GGTCGACACCGTTCATCTGA	qRT-PCR
*AalRp49_real+*	AGAAGTTCCTGGTCCACAAC	qRT-PCR
*AalRp49_real−*	GTTCTGCTGCGAGCGCAG	qRT-PCR
*Aalfru_MB_T7+*	taatacgactcactatagggAGTTCTCAATAGTATGTCATCG	dsRNA synthesis
*Aalfru_MB_T7−*	taatacgactcactatagggGCGGTGCGATTGCAGTTTTC	dsRNA synthesis
*eGFP_T7+*	taatacgactcactatagggGGTGAACTTCAAGATCCGCC	dsRNA synthesis
*eGFP_T7−*	taatacgactcactatagggGCATGGACGAGCTGTACAAG	dsRNA synthesis

**Table 2:** Number of injected embryos and adult individuals obtained in RNAi knockdown.

Name	Injected Embryos	Larvae	Pupae	Adults	Males	Females	Larval Survival Rate (%)
*AalfruM dsRNA*	1375	22 *	9	9	8	1	1.60
*GFP dsRNA*	516	7 †	7	7	4	3	1.36

* Thirteen larvae were used to perform qRT-PCR analysis of *fru* expression level. † Three larvae were used to perform qRT-PCR analysis of *fru* expression level.

## Data Availability

Data are contained within the article and Supplementary Materials. The data presented in this study are available in Supplementary File S1.

## Supplementary Materials

The following supporting information can be downloaded at https://www.mdpi.com/article/10.3390/insects16030280/s1. File S1: *Aalfru* sequences.
