# Supplementary material for: The Early Sex-Specific Expression of the Fruitless Gene in the Asian Tiger Mosquito Aedes albopictus (Skuse) and Its Functional Conservation in Male Courtship
Source: Insects. 2025 Mar 7;16(3):280. doi: 10.3390/insects16030280 (PMC11943076; doi:10.3390/insects16030280)
Supplement: Supplementary file 1 [file insects-16-00280-s001.zip › insects-3509386-supplementary.pdf]

Supplementary Material

File S1 – Fasta file with sequences of the *Aalfru* gene produced in the present study. Length and primer pair names utilized for the PCR amplification of the cDNA fragments are indicated in square brackets.

>FRUM [921bp - M2+/C3-]

```
AGTTCTCAATAGTATGTCATCGCCGCCGGCAATGCCCATCTACAGCCGCTACCCGGCATTGAACGG
CTACCCTCAGATGAACGGTGTCGACCCAGGTGAACCGATCGATTGTAGAAAACTGCAATCGCACCG
CAGGAACAGCACTGACACAGGAACAATGGACCAGCAGTATTGCTTACGCTGGAACAACCATCAGTC
CAACCTGACGACCGTGCTCAGAACCCTGCTGGAGGACGAGAAACTGTGTGATGTCACGCTCGCCTG
CGATAATGGAATCGTCAAAGCACATCAGGCGATACTGTCGGCGTGCAGTCCGTACTTTGAGCAGAT
CTTCGTCGAGAACAAACACCCGCATCCGATCATCTACCTGCGTGACGTCGAGGTCAGCGAGATGCG
CGCCCTGCTCAACTTCATGTACCAGGGCGAGGTGAACGTCGGTCAGCACAACCTGCAGAACTTCCT
CAAGACGGCGGAGAGCTTAAAAGTACGAGGTCTCACCGAGAGCAACGCCGACCGGTACGCCACAGA
AGCGGAAAAAAGTCGAACCGAGCGGTCGAGAGTTGATTCGCGAGATGGACGTGACTCGGCACCGCC
CCCGGCCAGTGTCACCAACAACAACAACACCATCAACAGTAACAACAACAACAACAGTACTAATAA
CAACAACAACAACAATACTCTCCACCATCCGCTGCAGCGGGACAAAGAACTTCGTGAACGGGAAGA
GCTGAGGGAGCGGGAAAGGCGGGAAGTCCATCGGGAGCTGCAGAGAGAGCGGGAACTGCAGGCGCG
GGAACACCAGCGCAGTGCTAGTGCCGATCTGCTCACGCCGATGACCGACGACTGCCGGTACTCGCC
CTCGGACGACCGAGACTTGAACGTAGACAGTAAGAAGAAGCGGAAAATATCCACCTGTGATAACTC
GTTGCCCTCGACGCCGACCCTGATGAACGACCGGCCGGGTGGATACGAATCGCAGGT
```

>FRUF [2154bp - M2+/C3-]

```
AGTTCTCAATAGTATGTCATCGCCGCCGGCAATGCCCATCTACAGCCGCTACCCGGCATTGAACGG
CTACCCTCAGATGAACGGTGTCGACCCAGGTGAACCGATCGATTGTAGAAAACTGCAATCGCACCG
CAGGTATGTCCAGCGTGGTTTCCACAGGAAGAAAGTTAAGGCGAAAAACCGCCTCCCGTAATACAA
CTAATGATTCTACTAACATCCATCACGTTCTGTCGAGATCGTGTCCTGTACATTTACCATGCGAGC
TTGAATCAAGGGAAACACACATATGTATAAACAAATGAAATGATAACTCTTGAATTGATTTTACCT
TACGTAAAATTAACAAATCAGCAACGCCAGCCGTCTAACAGAGGAGAGAAATCAAAACCAACCTGC
AAACATTTGATATCCTGTAAAAAGAAACAAAAAGCAGTACTAATAGGCAAATACTATAATAATCAA
TGATCATAACGTCGAACGTAGCAGCAATTCCGGTTAACCGTTTTATCAAAAAATATATTTAAATAC
ACATAAAAAAGTAGCAAAGTGAGAAATAAAAGACATATCTTAACCCGTTCATCCTGGAAATAGTAT
ACAATGCGAGGTATACGCGCGAGAACTGTAAAAAGTTGTAAACAATCGAGCTGACAAATATTGCTT
ATCATTTTGAAGTATATTCCTGAAACAGACTAACTGTAAGTGCACAGAAACATTTTCCTCTTCGGT
AGTGTTTCGTCAATCAAAACGCGGTAGTCAAACGCTTTGCTTCCCGTAGTTATATTCGCAAGTTAA
TCATTAAAAGCAAAAAGTTAACCCAACCAATGCAAAAACCATTAACAAGACATGTAAATGATCAGT
AGAGTTTCCGAGTACAAAACGAACTTAGCTAAACCAAATCAAGGAATGAAATGCGAACTAACAGTG
AATCATATAGCATCATTTAGCCCGATTTATTAACAATATGCCCTAGGATCGTAATTTGTAAATTAA
ACCAAACTATTTAACCTGTTCCAAAACAAACCTTAAAAACTAAACACCCCAAACTCCCCGCGTACA
```

ATTGATCGATTCAACCAACTAAACCAACCCACCGACGACCACCGTAGTAACTATGTGATGCTTCTC
TTGCAACGAACAACAAACGAATACTGGCAACGCTTCTGCACTCGTCGCATCCTGCTGTCATCAACC
TACTATACCGTCACCGAGCATCCGAAACGCTGCCGGCCGGCTTAGCCGGAACACAAGGTGGCGGCC
GTGCCGGCTCAACAAACAGCAACCCAACCGCAAAGCGTTGTCCAGGAAAGCTGGGTGCTCTGCCAC
TCCAGTCAACGGGGGATTCACAGCGTTCGCCACCAACAATCGCTGCTGGAACAGCACTGACACAGG
AACAATGGACCAGCAGTATTGCTTACGCTGGAACAACCATCAGTCCAACCTGACGACCGTGCTCAG
AACCCTGCTGGAGGACGAGAAACTGTGTGATGTCACGCTCGCCTGCGATAATGGAATCGTCAAAGC
ACATCAGGCGATACTGTCGGCGTGCAGTCCGTACTTTGAGCAGATCTTCGTCGAGAACAAACACCC
GCATCCGATCATCTACCTGCGTGACGTCGAGGTCAGCGAGATGCGCGCCCTGCTCAACTTCATGTA
CCAGGGCGAGGTGAACGTCGGTCAGCACAACCTGCAGAACTTCCTCAAGACGGCGGAGAGCTTAAA
AGTACGAGGTCTCACCGAGAGCAACGCCGACCGGTACGCCACAGAAGCGGAAAAAAGTCGAACCGA
GCGGTCGAGAGTTGATTCGCGAGATGGACGTGACTCGGCACCGCCCCCGGCCAGTGTCACCAACAA
CAACAACACCATCAACAGTAACAACAACAACAACAGTACTAATAACAACAACAACAACAATACTCT
CCACCATCCGCTGCAGCGGGACAAAGAACTTCGTGAACGGGAAGAGCTGAGGGAGCGGGAAAGGCG
GGAAGTCCATCGGGAGCTGCAGAGAGAGCGGGAACTGCAGGCGCGGGAACACCAGCGCAGTGCTAG
TGCCGATCTGCTCACGCCGATGACCGACGACTGCCGGTACTCGCCCTCGGACGACCGAGACTTGAA
CGTAGACAGTAAGAAGAAGCGGAAAATATCCACCTGTGATAACTCGTTGCCCTCGACGCCGACCCT
GATGAACGACCGGCCGGGTGGATACGAATCGCAGGT

>FRUC-ZNFA [685 bp - C5+/ZnfA-]

ACATGCCTCCGCTGAACGACAGTGGCGATAACGAGGGCTCCCTCTCGCATCCCGACCACCCGGACA
ACATCGACGGTGACCGAATGTTGCGCCTGGTAATGGCGACCCGGCACCATCCTGGACTCTTACACC
ACGACTCGAGCGGAATGCCCGGCGGAAGCGGCGTTGGCGAAGGCCCACCCAACACCCCGGGAAGTC
TCCTGGAGCAGCACCACCACCTGAACGGGATCAGCCACCAGCAGTCGTCCACGTCTTCGCAACTGT
CGTCCAGCACTCCACCGGGAACCAGTTCCAGCATCGACCACTGCGGTAGCCTTTCAGTGCCAACCG
GTGGGAGTCCCGGTGGCGGCGGCGCCACCGGTTGCCCCCGCAGCGACGGCAGCAGTCGTTCGTCCA
GTTCGACCAGGCGCGACCACAACATTAACTACCCTTCGCTGTTCACCCAGTTGAACCGAACCTTCC
CAACCCTGTACAGCTGCGTCAGTTGCCACAAAACCGTCTCGAACCGTTGGCACCATGCCAACCTCC
AACGTCCGCAGAGCCACGAGTTCTCCGTGTGCGGCCAGAAGTTCACGCGCCGACACGACATGAAAG
CTCACTGCAAGATCAAACATCCGAACTAAGTGACCGTTTCTACAACCATCGAGTACACATGTAATA
TCGCTGTGCCCGGAACAAACAACGG

>FRUC-ZNFB [662bp - C5+/ZnfB-]

ACATGCCTCCGCTGAACGACAGTGGCGATAACGAGGGCTCCCTCTCGCATCCCGACAAACCGGACA
ACATCGACGGCTCCAAGGCCTGGCATATGCGGCTAACCTTTGAACGTCTATCGGGCGGCTGTAATC
TGCATCGCTGTAAACTTTGCGGCAAAGTTGTGACGCACATACGCAACCACTATCACGTGCACTTTC
CTGGCCGCTTCGAATGTCCGCTGTGTCGGGCCACCTACACCCGGAGCGATAACCTGCGGACGCACT
GCAAGTTCAAGCACCCGATGTTCAATCCGGACACGCGAAAATTCGATAATAACATGATGTCTCCGG
CGATGGTGTCCCAAGCGGCGGCAGCAGCGGCCGCCATATCGGCGGCTACGGCCGCTGCCAACAGCT
TCAAGCCCGATTTCTCCGCCGTGCCCAAAAGTTTCAAGTCGAAGCTGAACGCGGCGCCTGCGGCGG
CGCCCGCCAACCCCAAAAACGGTCTCAATCCTTTCAAGGCCGATTTTCCCATTCAAAATGCAAGGC
CGGCGGCCGCTGCGGCGGGGGGGGTTGCGGCTTCGGCCGCTTCCGCTTCCCCGGGAAAAAACCCCA

AGAGGGGAAACTCCGAATGAAAAAGGAAAAACCACTGGCCTCAATCGTACGGCCAGTCGAGCAGTG
CT

>FRUC-ZNFC [1186bp - C3+/ZnfC-]

GAGCGGTCGAGAGTTGATTCGCGAGATGGACGTGACTCGGCACCGCCCCCGGCCAGTGTCACCAAC
AACAACAACACCATCAACAGTAACAACAACAACAACAGTACTAATAACAACAACAACAACAATACT
CTCCACCATCCGCTGCAGCGGGACAAAGAACTTCGCGAACGGGAAGAGCAGAGGGAGCGGGAAAGG
CGGGAAGTCCATCGGGAGCTGGAGAGAGAGCGGGAACTGCAGGCGCGGGAACACCAGCGCAGTGCC
AGTGCCGATCTGCTCACGCCGATGACCGACGACTGCCGGTACTCGCCCTCGGACGACCGAGACTTG
AACGTAGACAGTAAGAAGAAGCGGAAAATATCCACCTGTGATAACTCGTTGCCCTCGACGCCGACC
CTGATGAACGACCGGCCGGGTGGATACGAATCGCAGGCGTCATCACACAGTAGTATCAAGCTAAGT
CCAAAGCCAGACGAAGAGTTCAAAGTCAGCTCGCCGGCCCCGATGCATTCGCTCGCCAGTCAGATC
AAACAGGAGTACTCCGACCTGCCCAGCCGTCATCACCCGATGCATCCAGAGTTGTTGCCTCCCATG
CCAATGAACCTCAACCCGGAGGAGATGAGCAACATGCTATCGCAGGGCAACATGCCTCCGCTGAAC
GACAGTGGCGATAACGAGGGCTCCCTCTCGCATCCCGACCACCCGGACAACATCGACGGTCCGGGG
GGAGGGCAGTATGAGCACCAATTAAACCATCACGCCAGCAGCATTCTCACGTCCTGTCTTGTAACG
TCGCCCGACGGACCCAGCGGAGGCGGCGGAGGCGGAGGCGCTGGTGATCTGTCCCTGCATCCGCAC
TTTCCGCCGCACCACCAGATGTCCTATCACAACATGTTCACCCCCTCGCGGGAACCGGGCACGGCC
TGGCGGTGCCGATCGTGCGGCAAGGAGGTCACCAACCGCTGGCATCACTTCCACTCGCACACCCCG
CAGCGATCCGTGTGCCCGTACTGTCCAGCGTCCTACAGCCGAATAGACACGCTCCGGTCGCATCTC
CGGTCGAAGCACGCCGACCGGCTGACGGCACCCCCCACACCCAAGTTCGGCACGTCGCCAAACTGC
AAAATGCAGATGTAAAACCTTCAGATCATCCGGATTACTACAGGGAACAAGGCGACACCTACCG
